# iNOS is not responsible for RyR1 S-nitrosylation in *mdx* mice with truncated dystrophin

**DOI:** 10.1186/s12891-020-03501-0

**Published:** 2020-07-21

**Authors:** Ken’ichiro Nogami, Yusuke Maruyama, Ahmed Elhussieny, Fusako Sakai-Takemura, Jun Tanihata, Jun-ichi Kira, Yuko Miyagoe-Suzuki, Shin’ichi Takeda

**Affiliations:** 1 Department of Molecular Therapy, National Institute of Neuroscience, National Center of Neurology and Psychiatry, Tokyo, Japan; 2 Department of Neurology, Neurological Institute, Graduate School of Medical Sciences, Kyushu University, Fukuoka, Japan; 3 Department of Neurology, Faculty of Medicine, Minia University, Minia, Egypt; 4 Department of Cell Physiology, The Jikei University School of Medicine, Tokyo, Japan; 5 National Center of Neurology and Psychiatry, Tokyo, Japan

**Keywords:** iNOS, nNOS, Duchenne muscular dystrophy, Becker muscular dystrophy, Ryanodine receptor 1 (RyR1)

## Abstract

**Background:**

Previous research indicated that nitric oxide synthase (NOS) is the key molecule for S-nitrosylation of ryanodine receptor 1 (RyR1) in DMD model mice (*mdx* mice) and that both neuronal NOS (nNOS) and inducible NOS (iNOS) might contribute to the reaction because nNOS is mislocalized in the cytoplasm and iNOS expression is higher in *mdx* mice. We investigated the effect of iNOS on RyR1 S-nitrosylation in *mdx* mice and whether transgenic expression of truncated dystrophin reduced iNOS expression in *mdx* mice or not.

**Methods:**

Three- to 4-month-old C57BL/6 J, *mdx*, and transgenic *mdx* mice expressing exon 45–55-deleted human dystrophin (Tg/*mdx* mice) were used. We also generated two double mutant mice, *mdx* iNOS KO and Tg/*mdx* iNOS KO to reveal the iNOS contribution to RyR1 S-nitrosylation. nNOS and iNOS expression levels in skeletal muscle of these mice were assessed by immunohistochemistry (IHC), qRT-PCR, and Western blotting. Total NOS activity was measured by a citrulline assay. A biotin-switch method was used for detection of RyR1 S-nitrosylation. Statistical differences were assessed by one-way ANOVA with Tukey-Kramer post-hoc analysis.

**Results:**

*mdx* and *mdx* iNOS KO mice showed the same level of RyR1 S-nitrosylation. Total NOS activity was not changed in *mdx* iNOS KO mice compared with *mdx* mice. iNOS expression was undetectable in Tg/*mdx* mice expressing exon 45–55-deleted human dystrophin, but the level of RyR1 S-nitrosylation was the same in *mdx* and Tg/*mdx* mice.

**Conclusion:**

Similar levels of RyR1 S-nitrosylation and total NOS activity in *mdx* and *mdx* iNOS KO demonstrated that the proportion of iNOS in total NOS activity was low, even in *mdx* mice. Exon 45–55-deleted dystrophin reduced the expression level of iNOS, but it did not correct the RyR1 S-nitrosylation. These results indicate that iNOS was not involved in RyR1 S-nitrosylation in *mdx* and Tg*/mdx* mice muscles.

## Background

Duchenne muscular dystrophy (DMD) is an X-linked genetic disease characterized by progressive muscle weakness due to a lack of dystrophin [1]. DMD is caused by frame-shift deletions or nonsense mutations in the *DMD* gene. Becker muscular dystrophy (BMD), in which the reading frame in the *DMD* gene is not altered, is similar to DMD, but the progression of symptoms is slower and less severe than DMD because BMD patients have truncated but partially functional dystrophin [2]. In dystrophic muscle, the sarcolemma is easily ruptured by mechanical stresses, such as muscle contraction, and Ca^2+^ flows into the cytoplasm. Intracellular Ca^2+^ overload leads to muscle contracture, mitochondrial dysfunction, and activation of proteases. These are the key factors of muscle degeneration and necrosis in DMD [3]. In addition, Ca^2+^ regulation in the sarcoplasmic reticulum (SR) is impaired in dystrophic muscle, and this is also related to DMD pathogenesis [4]. Ryanodine receptor 1 (RyR1), which releases Ca^2+^ from SR to the cytoplasm, is important for muscle contraction. In DMD model mice (*mdx* mice), RyR1 becomes leaky, because it is S-nitrosylated by nitric oxide synthase (NOS) [4]. NO is known as a key regulator of many proteins by S-nitrosylation of cysteine residues [5, 6]. Bellinger et al. showed that RyR1 is S-nitrosylated in *mdx* muscle and that inducible NOS (iNOS) plays an important role in this reaction [4].

Recent studies, however, showed that neuronal NOS (nNOS), which is one of the constitutional types of NOS, is responsible for RyR1 S-nitrosylation [7, 8]. nNOS usually exists on the sarcoplasm with dystrophin. It binds to α1-syntrophin and also directly binds to the rod domain of dystrophin (spectrin-like repeats 16 and 17), but it is mislocalized and activated in the cytoplasm when the muscle lacks dystrophin protein, which causes RyR1 S-nitrosylation [7–12]. Another report showed that iNOS was not responsible for RyR1 S-nitrosylation by using iNOS KO-*mdx*4cv double mutant mice, which is another DMD model mouse [13]; therefore, which NOS isoform is responsible for RyR1 S-nitrosylation is still controversial.

Previously, we generated transgenic *mdx* mice expressing exon 45–55-deleted human dystrophin (Tg/*mdx* mice) to confirm the underlying molecular mechanisms of truncated dystrophin. We found that nNOS was still mislocalized in Tg/*mdx* mice and RyR1 S-nitrosylation was not changed, because Tg/*mdx* mice have partially functional dystrophin, but lack a part of the nNOS binding site which is encoded by exons 42–45 [9, 14]. It has been, however, still unknown which NOS isoforms are responsible for the RyR1 S-nitrosylation in Tg/*mdx* mice. In this study, we generated two double-mutant mice, *mdx* iNOS KO and Tg/*mdx* iNOS KO, to study further the mechanism of RyR1 S-nitrosylation with nNOS and iNOS.

We revealed that *mdx* and *mdx* iNOS KO mice showed the same level of RyR1 S-nitrosylation. Interestingly, these mice also had the same level of total NOS activity. This suggests that the proportion of iNOS in total NOS activity was low even in *mdx* mice. iNOS expression was suppressed and undetectable in Tg/*mdx* mice, although RyR1 S-nitrosylation was not changed.

Taken together, our results indicate that nNOS rather than iNOS is responsible for S-nitrosylation of RyR1 in *mdx* and Tg/*mdx* mice.

## Methods

### Animals

**Fig. 1:**
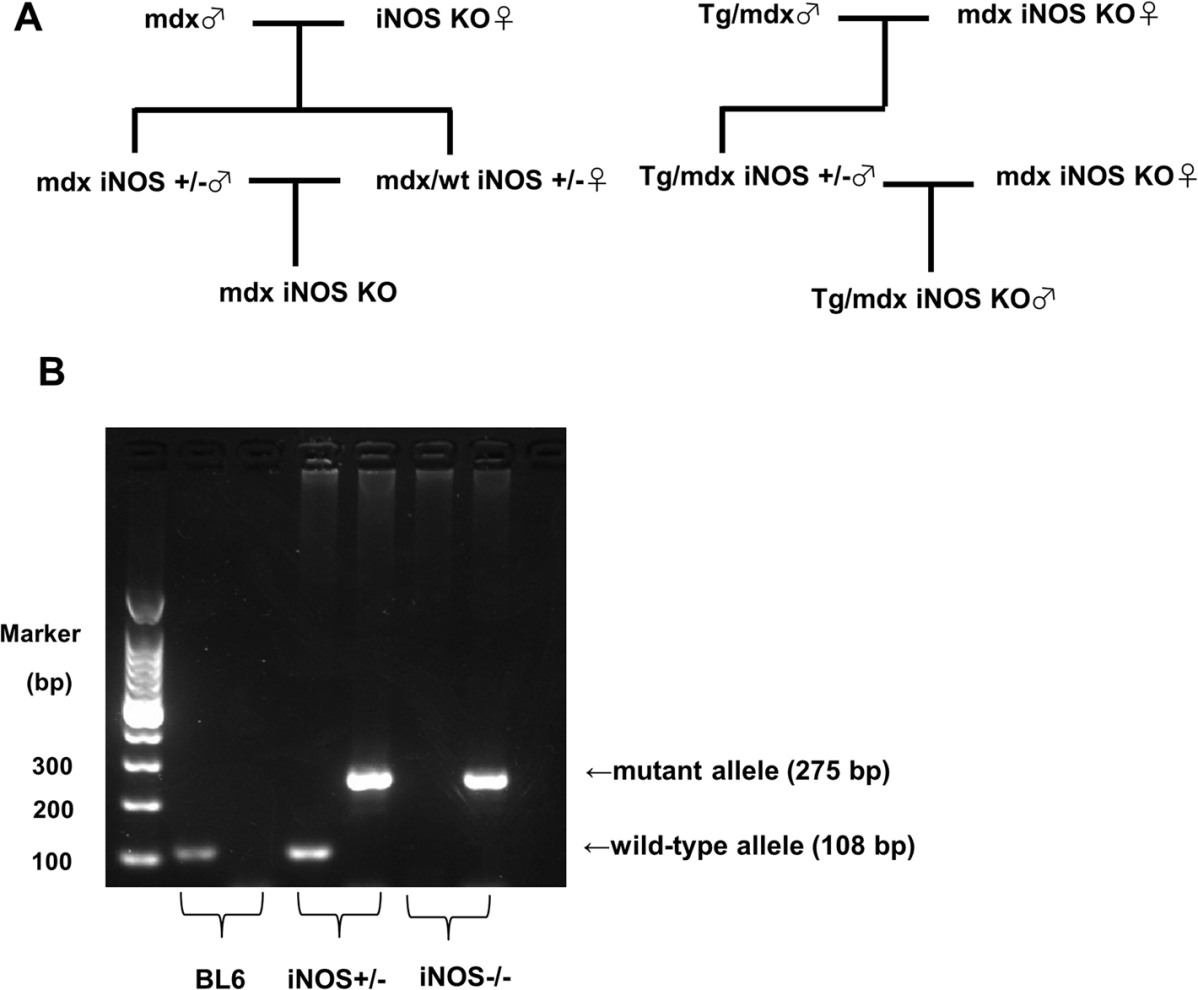
Generation of two double-mutant mice: *mdx* iNOS KO and Tg/*mdx* iNOS KO mice. **a** The breeding scheme of *mdx* iNOS KO and Tg/*mdx* iNOS KO mice. **b** Determination of iNOS gene mutation by PCR. The PCR product of the wild type allele is 108 bp and that of the knockout (mutant) allele is 275 bp

Transgenic *mdx* mice expressing exon 45–55-deleted human dystrophin (Tg/*mdx*) were obtained as previously described [14]. C57BL/6 J (BL6), *mdx*, and iNOS KO mice with a C57BL/6 J background were purchased from Nihon CREA (Tokyo, Japan) and Jackson Laboratory (Bar Harbor, ME). *mdx* iNOS KO-double mutant mice were generated by crossing iNOS KO and *mdx* mice (Fig. 1a). The genotype of *mdx* mice was determined by primer competition PCR as reported by Shin et al. [15]. The genotype of iNOS KO was determined as described by Li et al. [13] (Fig. 1b). Tg/*mdx*-iNOS KO mice were generated by crossing Tg/*mdx* mice and *mdx* iNOS KO mice (Fig. 1a). The experimental mice were 3–4 months old. Only male mice were used in the study. Mice were bred at the specific pathogen-free (SPF) animal facility in the National Institute of Neuroscience, NCNP, and were allowed free access to food and drinking water. The Experimental Animal Care and Use Committee of the National Institute of Neuroscience of the NCNP approved all experimental protocols in this study (Approval ID: 2018041).

### Antibodies

Rabbit polyclonal antibody against dystrophin (#ab15277), nNOS (#61–7000), and iNOS (#482728) were purchased from Abcam (Tokyo, Japan), Invitrogen (Carlsbad, CA) and Sigma-Aldrich (St. Louis, MO), respectively. Rat monoclonal antibody against F4/80 (#T2028) was purchased from BMA Biomedicals (Augst, Switzerland). Mouse monoclonal antibody against RyR1 (R129) was purchased from Sigma-Aldrich (St. Louis, MO). Goat polyclonal antibody against GAPDH (V-18) was purchased from Santa Cruz (Santa Cruz, CA, USA). Rabbit polyclonal antibody against α1-syntrophin [16] was a kind gift from Dr. Michihiro Imamura (National Center of Neurology and Psychiatry).

### Tissue preparation

Mice were sacrificed by cervical dislocation. The tibialis anterior (TA) and gastrocnemius (GC) and diaphragm (DIA) muscles were collected using standard dissection methods. Muscles were frozen in isopentane cooled by liquid nitrogen for histological analysis, RNA, or protein isolation. All samples were stored at − 80 °C.

### Immunohistochemistry, histology

Cryosections were cut from TA and DIA muscles at 8 μm and stained with hematoxylin and eosin (H&E). Immunohistochemistry was performed as described previously [17]. In brief, sections were fixed in cold acetone and incubated in TBS containing 0.1% Triton-100 for 1 min at room temperature. The sections were washed and stained with primary antibodies in TBS containing 2% casein overnight at 4 °C, followed by incubation with Alexa 488-conjugated goat anti-rabbit IgG antibody or Alexa 594-conjugated goat anti-rat IgG antibody (Invitrogen). Fluorescence images were obtained using a BZ-X810 fluorescence microscope (Keyence, Osaka, Japan).

### RNA isolation and qRT-PCR analysis

RNA isolation from TA muscles and cDNA synthesis were performed as previously described [18]. Expression levels of mRNA were measured by quantitative RT-PCR (qRT-PCR) using the SYBR Premix Ex TaqII (Takara). Primer sequences for qRT-PCR were as follows: iNOS forward, 5′- TGACCATCATGGACCACCAC-3′, reverse, 5′- ACCAGCCAAATCCAGTCTGC-3′; nNOS forward, 5′- ACCAGCACCTTTGGCAATGGAG-3′, reverse, 5′- GAGACGCTGTTGAATCGGACCT -3′; GAPDH forward, 5′- GTGAAGGTCGGTGTGAACG − 3′, reverse, 5′- CAATCTCCACTTTGCCACTG − 3′. The expression levels of these genes were normalized to those of GAPDH.

### Western blot analysis

Total protein from TA muscles was extracted by a sample buffer containing 15% glycerol, 1 mM dithiothreitol, 2% SDS, 125 mM Tris-HCl, and protease inhibitor cocktails cOmplete. Protein lysate was then incubated at 100 °C for 5 min and centrifuged at 10,000 rpm for 5 min. The supernatant was used for SDS-polyacrylamide gel electrophoresis (SDS-PAGE). The protein concentration was determined using a protein assay (Bio-Rad Laboratories, Inc., Hercules, CA) with bovine serum albumin as a standard in an SDS concentration that does not affect the accuracy of the assay system. The samples were separated on an SDS-polyacrylamide gel and electrically transferred from the gel to a polyvinylidene difluoride membrane (Millipore, Darmstadt, Germany). The blot was incubated with primary antibodies. The signals were detected using the ECL Prime Western Blotting Detection Reagent (GE Healthcare, UK, Ltd.; RPN2232) and a ChemiDoc MP Imaging System (Bio-Rad). Data were analyzed using Image Lab 6.0 (Bio-Rad).

### Biotin switch assay

To detect the S-nitrosylation of RyR1, a biotin switch assay was carried out based on a modified procedure described previously [19]. Total protein from GC muscles was homogenized in HENS buffer containing 0.5% (w/v) CHAPS, 0.1% (w/v) SDS, 20 mM NEM, cOmplete protease inhibitor cocktail, and calpain inhibitor I and kept on ice for 30 min to block sulfhydryl groups. The supernatant was supplemented with SDS at a final concentration of 1% (w/v), and incubated for 30 min at RT. Excess NEM was removed by protein precipitation with acetone, and the pellet was resuspended and incubated for 1 h at RT in HENS buffer containing 1% (w/v) SDS, 10 mM sodium ascorbate, and S-Nitrosylation Labeling reagents in the kit as per manufacturer’s instructions (Cayman Chemical, Ann Arbor, MI, USA) for reduction of S-nitrosothiols and labeling with biotin. Extra labeling was removed by a second acetone precipitation. Proteins were resuspended in lysis buffer containing 25 mM Tris-HCl, pH 7.5, 100 mM NaCl, 2 uM EDTA, 1% (v/v) Triton-X-100, 0.1% (w/v) SDS, cOmplete protease inhibitor cocktail, and calpain inhibitor I. To pull down SNO proteins, streptavidin-conjugated magnetic beads (2.8 um, Dynal magnetic beads; Invitrogen Life Technologies Corp., Carlsbad, CA, USA) were used. The beads were washed 3 times with lysis buffer and added to the biotin-labeled samples. The mixture was rotated at room temperature for 1 h. After removing the supernatant by magnetic separation, SNO proteins were eluted in 50 μL of SDS-loading buffer. Protein was separated by SDS-PAGE and electroblotted onto a PVDF membrane. Membranes were blocked in TBST containing 3% bovine serum albumin for 1 h at RT. Protein was detected by immunoblotting using polyclonal antibody against RyR1 (Sigma-Aldrich; R129). S-nitrosylated RyR1 was normalized by the intensity of the RyR1 signal of whole muscle lysate.

### NOS activity assay

NOS activity was determined by the citrulline assay as previously described [20] using the NOS assay kit (Cayman Chemical). Fresh quadriceps muscles were homogenized in 5 volumes of buffer containing 25 mM Tris-HCl (pH 7.4), 1 mM EDTA, and 1 mM EGTA. The homogenate was centrifuged at 10000 g for 15 min at 4 °C. The Supernatant was mixed with a reaction mixture contained 25 mM Tris-HCl, pH 7.4, 3 μM tetrahydrobiopterin, 1 μM FAD, 1 μM FMN, 1 mM NADPH, 600 μM CaCl_2_, 0.1 μM calmodulin, and 1 μCi [^3^H] Arg (Amersham Biosciences, Bucks, UK). After incubation for 30 min at 37 °C, the reaction was stopped by adding stop buffer (50 mM HEPES, pH 5.5, 5 mM EDTA). A resin slurry was added to the reaction mixture, and the resin was removed by centrifuging. The flow-through containing [^3^H] citrulline was added to the scintillation liquid, and radioactivity was counted. Particularly, iNOS activity was determined using a reaction mixture containing MgCl_2_ instead of CaCl_2_ and incubating 2 h at 37 °C.

### Statistical analysis

All values are expressed as means ± SEM. Statistical differences were assessed by a one-way ANOVA with Tukey-Kramer post-hoc analysis. Probabilities less than 5% (*P < 0.05), 1% (**P < 0.01), 0.1% (***P < 0.001) or 0.01% (****P < 0.0001) were considered to be statistically significant.

## Results

### Transgenic expression of exon 45–55-deleted human dystrophin reduced iNOS expression in *mdx* mice

A previous report showed that somatic gene transfer of dystrophin or utrophin reduced iNOS expression in *mdx* mice [21]. Another report also described the reduction of iNOS expression of iNOS by exon skipping treatment in golden retriever muscular dystrophy dogs [22]. It is, however, still unknown whether truncated dystrophin could prevent iNOS upregulation in *mdx* mice. To study the effect on the expression of iNOS by truncated dystrophin, we used transgenic *mdx* mice expressing exon 45–55-deleted human dystrophin (Tg/*mdx* mice). We also produced *mdx* iNOS KO and Tg/*mdx* iNOS KO mice to reveal the role of iNOS in *mdx* and Tg/*mdx* mice.

**Fig. 2:**
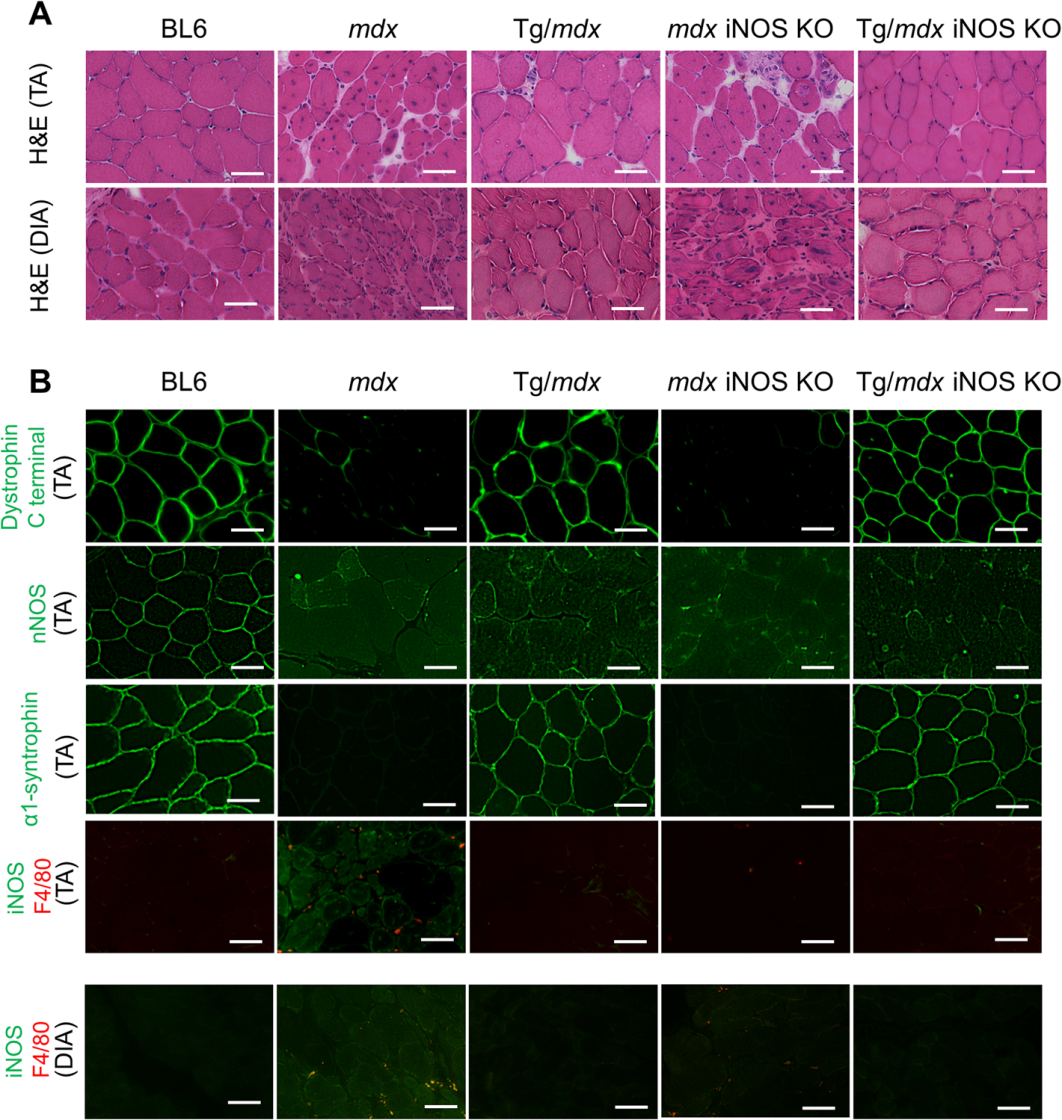
Expression of iNOS was detected in *mdx* muscle and reduced in Tg/*mdx* muscle. **a** H&E staining of TA and DIA muscles. **b** Immunohistochemically staining of dystrophin (green), nNOS (green), α1-syntrophin (green), iNOS (green), and F4/80 (red) of TA and DIA muscles. The experimental mice were 3–4 months old. Scale bar 50 μm

As previously described, exon 45–55-deleted dystrophin rescued membrane stability in Tg/*mdx* mice [14]; further, no degeneration or inflammatory cell infiltration into skeletal muscle was observed in Tg/*mdx* or in Tg/*mdx* iNOS KO mice although *mdx* and *mdx* iNOS KO mice both had necrotic fibers (Fig. 2a). Immunohistochemistry showed restored dystrophin and α1-syntrophin expression on the sarcolemma in Tg/*mdx* and Tg/*mdx* iNOS KO mice. nNOS was abnormally localized in the cytoplasm in *mdx* and *mdx* iNOS KO mice due to a lack of dystrophin. Tg/*mdx* and Tg/*mdx* iNOS KO mice also showed nNOS mislocalization because they expressed partially functional dystrophin, but it lacked the part of the nNOS binding site that is encoded by exons 42–45 [9, 14]. The iNOS signal was detected only in *mdx* mice, and was undetectable in Tg/*mdx* mice, suggesting that the truncated dystrophin almost completely suppressed the expression of iNOS. *mdx* iNOS KO and Tg/*mdx* iNOS KO mice did not show any iNOS signal, which suggested that we achieved a knockout of iNOS in these models (Fig. 2b). Diaphragm muscle of *mdx* mice shows severe histopathological features and the expression of iNOS was detected in diaphragm muscle of *mdx* mice; however, it was also suppressed by truncated dystrophin (Fig. 2a, b).

### mRNA and protein expression of iNOS was suppressed in Tg/*mdx* mice and weak even in *mdx* mice

**Fig. 3:**
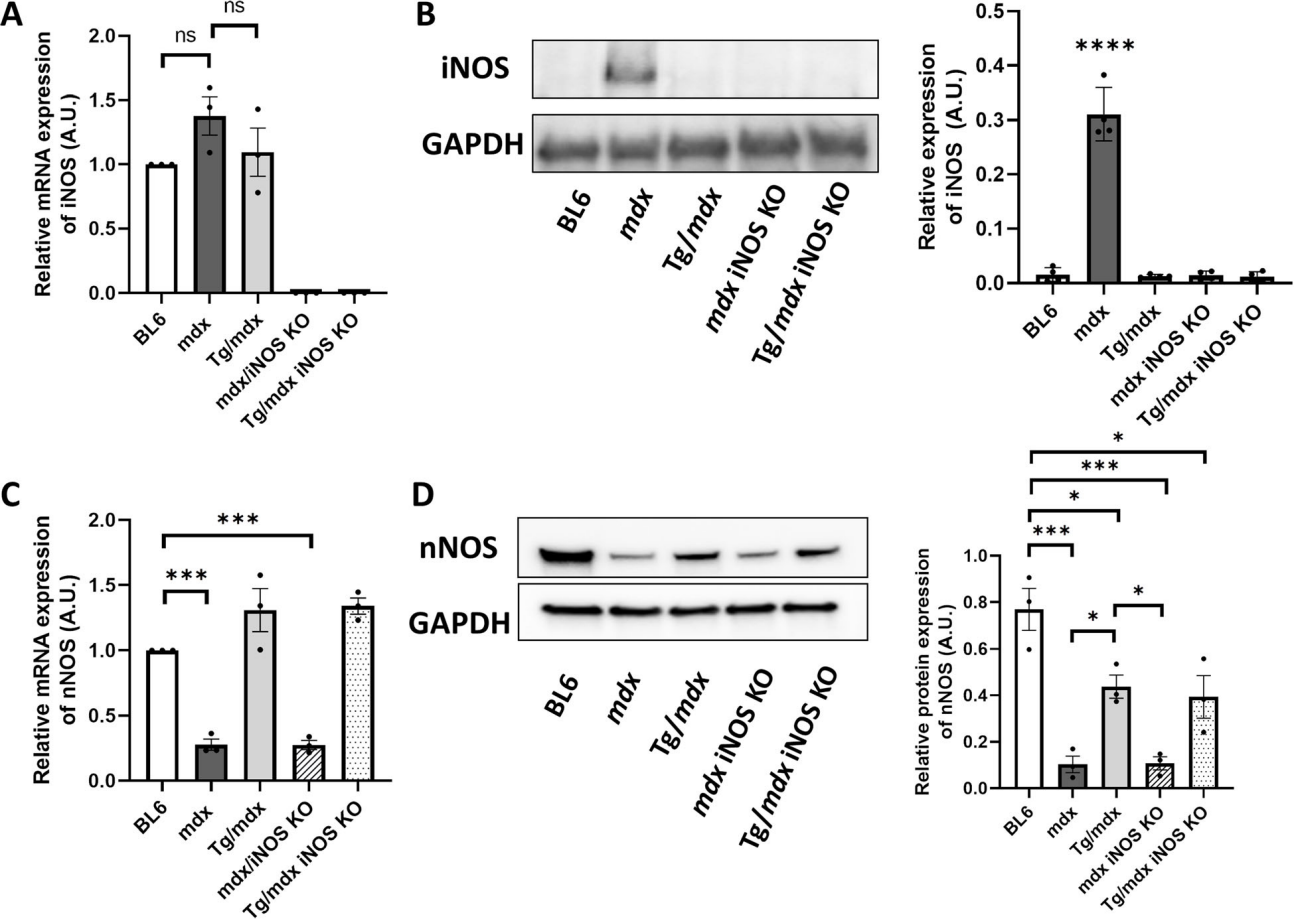
Protein expression of iNOS was detected only in *mdx* mice. **a** Quantification of qRT-PCR products for iNOS expression in TA muscles. **b** Western blots and quantification of iNOS in TA muscles relative to the GAPDH. **c** Quantification of qRT-PCR products for nNOS expression in TA muscles. **d** Western blots and quantification of nNOS in TA muscles relative to the GAPDH. The original full blot for (**b**) and (**d**) with a loading control are shown in Additional file 2 and 3, respectively. Data are presented as means ± SEM. *p < 0.05, ***p < 0.001, ****p < 0.0001 by ANOVA with Tukey-Kramer test (*n* = 3 mice per group)

Then, we assessed the mRNA expression of iNOS by qRT-PCR. We designed an iNOS primer inside the region of exons 12 and 13 of the iNOS gene because iNOS KO mice lacked this part of the exon. Surprisingly, mRNA expression of iNOS was very weak even in *mdx* mice, and it was almost same level as that of BL6 mice. Tg/*mdx* mice also did not show mRNA expression of iNOS (Fig. 3a). We confirmed that our iNOS primer worked by using RAW264.7 cells with LPS as a positive control (data not shown). This result suggested that iNOS mRNA expression in skeletal muscle was very low even in *mdx* mice. We detected protein expression of iNOS in tibialis anterior muscle of *mdx* mouse by Western blotting, and it was significantly higher than BL6 mice (Fig. 3b). Tg/*mdx* mice did not show protein expression of iNOS at all. We also assessed protein expression of iNOS in diaphragm muscle of each mouse, and the signal was detected only in *mdx* muscles (see Additional file 1). The original full blot of iNOS for both tibialis anterior and diaphragm muscle with a loading control are shown in Additional file 2. These results suggested that iNOS expression was detectable in *mdx* mice, but exon 45–55-deleted truncated dystrophin, which restores membrane stability and prevents muscle degeneration, completely suppressed the expression of iNOS. To confirm the effects of iNOS KO on the nNOS expression, we also checked mRNA and protein levels of nNOS. mRNA expression of nNOS was significantly lower in *mdx* mice, but Tg/*mdx* mice showed the same mRNA expression level of nNOS as BL6 mice. mRNA expression of nNOS did not change in *mdx* iNOS KO and Tg/*mdx* iNOS KO mice compared with *mdx* and Tg/*mdx* mice, respectively (Fig. 3c). In Western blotting, *mdx* mice showed lower protein expression of nNOS than BL6 mice. The protein level of nNOS was also lower in Tg/*mdx* mice, but partially restored compared with that of *mdx* mice. iNOS KO also did not affect to the expression of nNOS in *mdx* iNOS KO and Tg/*mdx* iNOS KO mice (Fig. 3d). The original full blot of nNOS for Fig. 3d with a loading control is shown in Additional file 3.

### iNOS catalytic activity was suppressed in Tg/*mdx* mice

**Fig. 4:**
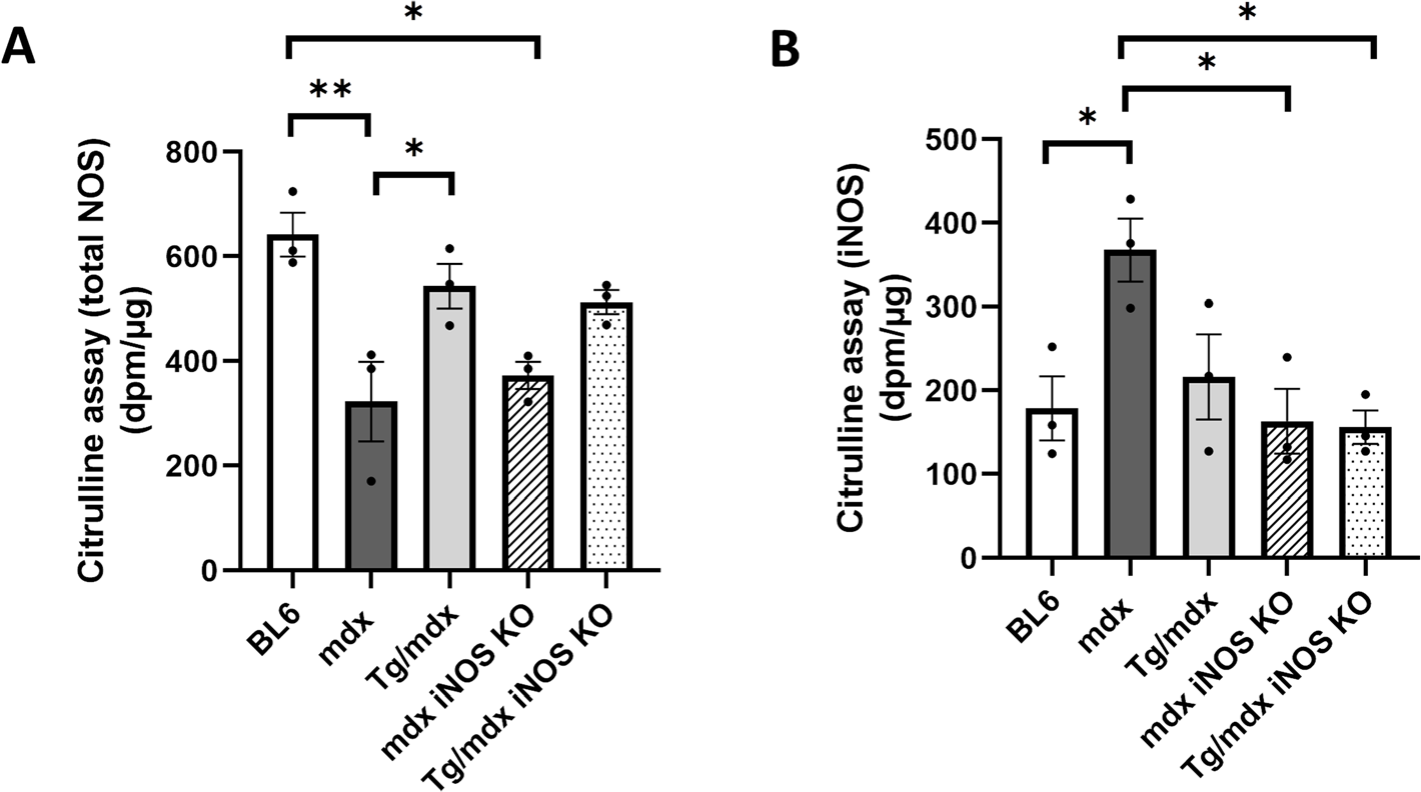
Total NOS activity was not changed by iNOS KO in *mdx* mice. **a** Total NOS and iNOS-specific catalytic activity in quadriceps muscles estimated by quantifying citrulline. Data are presented as means ± SEM. *p < 0.05, **p < 0.01 by ANOVA with Tukey-Kramer test (*n* = 3 mice per group)

Next, we confirmed the total NOS catalytic activity and iNOS-specific activity in each mouse by citrulline assay. The total NOS activity in freshly isolated quadriceps muscles from *mdx* mice was significantly lower than that in BL6 mice. On the other hand, Tg/*mdx* mice showed the same level of total NOS activity as BL6 mice (Fig. 4a). Interestingly, the total NOS activities in *mdx* and *mdx* iNOS KO mice were the same. It suggested that the proportion of iNOS in total NOS activity was quite low even though *mdx* mice expressed iNOS in skeletal muscle (Fig. 3b). We then assessed iNOS-specific activity. nNOS requires Ca^2+^ for its activity, but iNOS activity is independent of Ca^2+^ [23–25]. Therefore, we checked the iNOS-specific activity in a Ca^2+^-free condition. Recombinant iNOS as a positive control was strongly detectable by our method (data not shown), but it was difficult to detect iNOS-specific activity by using the lysate of skeletal muscle even from *mdx* mice. We modified the experimental protocol of the NOS activity assay (see Methods), and after a long reaction of samples with [^3^H] arginine, we detected the iNOS activity, but it was increased only in *mdx* mice (Fig. 4b). The iNOS activity in Tg/*mdx* mice did not show the significant decrease, but that in BL6, *mdx* iNOS KO, and Tg/*mdx* iNOS KO mice was significantly suppressed. This result suggested that iNOS activity was weak even in *mdx* mice and that nNOS may mainly contribute to the total NOS activity in skeletal muscle. This indication would be plausible because the total NOS activity was low in *mdx* mice, and *mdx* mice also showed reduced expression of nNOS (Fig. 3c, d).

### iNOS knock-out did not improve RyR1 S-nitrosylation in *mdx* and Tg/*mdx* mice

**Fig. 5:**
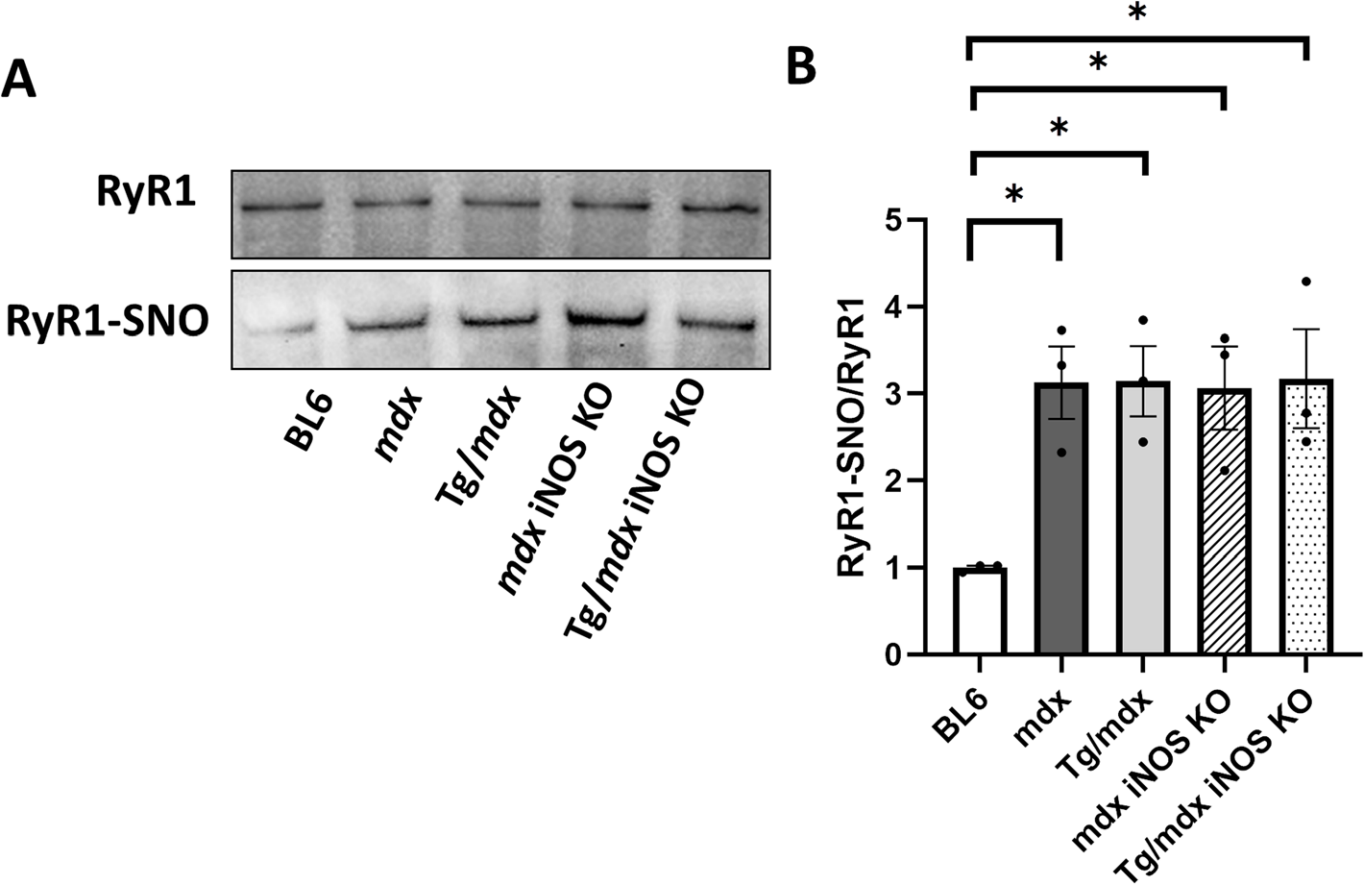
RyR1 S-nitrosylation was not changed in Tg/*mdx*, *mdx* iNOS KO, or Tg/*mdx* iNOS KO mice. **a** Western blots of RyR1 and S-nitrosylated RyR1 (RyR1-SNO) in GC muscles using a biotin-switch assay. **b** Quantification of relative expression of RyR1 S-SNO compared to those of total RyR1. The original full blot for (**a**) with a loading control is shown in Additional file 4. Data are presented as means ± SEM. *p < 0.05 by ANOVA with Tukey-Kramer test (*n* = 3 mice per group)

RyR1 is highly S-nitrosylated in *mdx* skeletal muscle compared with that of BL6 mice, as previously described [4]. On the other hand, which NOS isoform (nNOS, iNOS) was responsible for RyR1 S-nitrosylation was controversial [7, 13]. To reveal the role of iNOS in this reaction, we checked RyR1 S-nitrosylation by a biotin switch method. *Mdx* and *mdx* iNOS KO mice showed the same level of RyR1 S-nitrosylation. Tg/*mdx* did not show any difference in RyR1 S-nitrosylation compared with that of BL6 mice (Fig. 5a, b). The original full blot for Fig. 5a with a loading control is shown in Additional file 4. These results also indicate that the truncated dystrophin lacking a part of the nNOS-binding site did not rescue the RyR1 S-nitrosylation even though iNOS expression was suppressed in Tg/*mdx* mice (Fig. 3b). The same result was observed in Tg/*mdx* iNOS KO mice. These results reveal that iNOS was not responsible for RyR1 S-nitrosylation in *mdx* and Tg/*mdx* mice.

## Discussion

In this study, we revealed the relationship between iNOS and RyR1 S-nitrosylation in *mdx* mice and the transgenic *mdx* mice expressing exon 45–55-deleted human dystrophin (Tg/*mdx* mice) produced by Tanihata et al. [14]. We focused on the role of exons 45–55, because this segment of the DMD gene is the “hot spot” among DMD mutations, affecting about 60% of DMD patients [26]. Therefore, deletion of exons 45–55 would be the goal of exon skipping [27] or genome editing by CRISPR-Cas9 [28]. Previous reports showed patients who have the in-frame deletion of exons 45–55 of the *DMD* gene showed very mild phenotypes [26, 29]. In addition, a human clinical report showed that the variable phenotypes of patients with exon 45–55 deleted correlated with nNOS mislocalization and RyR1 S-nitrosylation [7]. Taken together, the role of nNOS in the phenotype of exon 45–55 deletion is important. The role of nNOS was recently shown by several researchers [7–12], but the role of iNOS in dystrophic muscle was still controversial [13, 30].

Bellinger et al. previously showed that iNOS was upregulated in *mdx* mice, and they concluded that iNOS was responsible for RyR1 S-nitrosylation because iNOS could be detected with RyR1 after co-immunoprecipitation and colocalized with RyR1 by immunohistochemistry [4]. The role of iNOS, however, became controversial after Li et al. showed that RyR1 S-nitrosylation was not altered in *mdx*4Cv iNOS KO double-mutant mice when compared with that of *mdx*4Cv mice [13]. To examine the participation of iNOS in *mdx* mice, we generated *mdx* iNOS KO double mutant mice. *Mdx* iNOS KO mice showed the same level of RyR1 S-nitrosylation as *mdx* mice. This result is consistent with the result of *mdx* 4Cv iNOS KO double-mutant mice [13]. Interestingly, *mdx* and *mdx* iNOS KO mice showed the same level of total NOS activity. It suggested that the proportion of iNOS in total NOS activity was low even in *mdx* mice. This result also strengthened the lack of responsibility of iNOS for RyR1 S-nitrosylation in *mdx* muscle. There is, however, a possibility that the participation of iNOS might be only at the earlier necrosis-degeneration stage of *mdx* mice around 2–4 weeks old [30, 31]. Villalta et al. showed iNOS protein levels were elevated in macrophages from 4-weeks-old *mdx* muscles, although they did not mention the expression of iNOS in skeletal muscle. In this study, we used 3–4 months old *mdx* mice because previous report showed iNOS levels were significantly increased in *mdx* muscles at 35 and 180 days of age [4].

We also revealed the relationship between iNOS expression and RyR1 S-nitrosylation in *mdx* mice expressing exon 45–55-deleted human dystrophin (Tg/*mdx* mice). The protein level of iNOS was suppressed and undetectable by the expression of exon 45–55-deleted dystrophin in *mdx* mice. iNOS expression was reduced because the truncated dystrophin was adequate to protect membrane stability and prevent muscle degeneration (Fig. 2a). *Mdx*, Tg/*mdx*, and Tg/*mdx* iNOS KO mice all showed the same level of RyR1 S-nitrosylation, which suggests that iNOS was not responsible for RyR1 S-nitrosylation, and that abnormal nNOS localization was the main factor for that reaction. Our results indicate the importance of nNOS in RyR1 S-nitrosylation, but the molecular mechanism of nNOS is not fully understood. Interestingly, the expression of α1-syntrophin was restored to the sarcolemma by truncated dystrophin, although nNOS was still localized in the cytosol (Fig. 2b). This result clearly shows the discrepancy between the sarcolemmal localization of α1-syntrophin and cytosolic expression of nNOS, indicating the role of spectrin-like repeats 16–17 of dystrophin for the sarcolemmal localization of nNOS. Further experiments are required to clarify why the abnormal distribution of nNOS in cytoplasm has a big effect on RyR1 S-nitrosylation, even though nNOS expression was lower in *mdx* mice.

## Conclusion

*Mdx* and *mdx* iNOS KO mice showed the same level of RyR1 S-nitrosylation. The proportion of iNOS in total NOS activity was low even in *mdx* mice. Transgenic expression of the exon 45–55-deleted human dystrophin reduced iNOS expression in *mdx* mice, but RyR1 S-nitrosylation still remained in Tg/*mdx* mice. These results suggested that iNOS is not involved in RyR1 S-nitrosylation in *mdx* and Tg/*mdx* mice muscles.

## Supplementary information

**Additional file 1 **Protein expression of iNOS in DIA muscles. Western blots and quantification of iNOS in DIA muscles relative to the GAPDH. The original full blot with a loading control is shown in Additional file 2. Data are presented as means ± SEM. ****p < 0.0001 by ANOVA with Tukey-Kramer test (*n* = 3 mice per group).

**Additional file 2.** The original full blot of iNOS for both tibialis anterior and diaphragm muscle. (A) Whole image of PVDF membrane of Fig. 3b (iNOS expression in TA muscle) and Additional file 1 (iNOS expression in DIA muscle) stained by Coomassie Brilliant Blue. The membrane was stained immediately after transferring. (B) Whole image of the immuno-Western blot of Fig. 3b and Additional file 1.

**Additional file 3.** The original full blot of nNOS. (A) Whole image of PVDF membrane of Fig. 3d (nNOS expression in TA muscle) stained by Coomassie Brilliant Blue. The membrane was stained immediately after transferring. (B) Whole image of the immuno-Western blot of Fig. 3d.

**Additional file 4.** The original full blot of RyR1 and S-nitrosylated RyR1. (A) Whole image of PVDF membrane of Fig. 5a stained by Coomassie Brilliant Blue. The membrane was stained immediately after transferring. (B) Whole image of the immuno-Western blot of Fig. 5a.

## Data Availability

The dataset analyzed in the current study is available from the corresponding author on reasonable request.

## Abbreviations

DMD: Duchenne muscular dystrophy
BMD: Becker muscular dystrophy
iNOS: inducible NOS
nNOS: neuronal NOS
RyR1: ryanodine receptor 1
SEM: standard error of the mean

## References

[1] Hoffman EP, Brown RH, Kunkel LM (1987). Dystrophin: the protein product of the Duchenne muscular dystrophy locus. Cell..

[2] Koenig M, Beggs AH, Moyer M, Scherpf S, Heindrich K, Bettecken T (1989). The molecular basis for Duchenne versus Becker muscular dystrophy: correlation of severity with type of deletion. Am J Hum Genet.

[3] Allen DG, Whitehead NP, Froehner SC (2016). Absence of dystrophin disrupts skeletal muscle signaling: roles of Ca2+, reactive oxygen species, and nitric oxide in the development of muscular dystrophy. Physiol Rev.

[4] Bellinger AM, Reiken S, Carlson C, Mongillo M, Liu X, Rothman L (2009). Hypernitrosylated ryanodine receptor calcium release channels are leaky in dystrophic muscle. Nat Med.

[5] Hess DT, Matsumoto A, Kim SO, Marshall HE, Stamler JS (2005). Protein S-nitrosylation: purview and parameters. Nat Rev Mol Cell Biol.

[6] Jaffrey SR, Erdjument-Bromage H, Ferris CD, Tempst P, Snyder SH (2001). Protein S nitrosylation: a physiological signal for neuronal nitric oxide. Nat Cell Biol.

[7] Gentil C, Leturcq F, Ben Yaou R, Kaplan JC, Laforet P, Pénisson-Besnier I (2012). Variable phenotype of del45-55 Becker patients correlated with nNOSμ mislocalization and RyR1 hypernitrosylation. Hum Mol Genet.

[8] Li D, Yue Y, Lai Y, Hakim CH, Duan D (2011). Nitrosative stress elicited by nNOSμ delocalization inhibits muscle force in dystrophin-null mice. J Pathol.

[9] Lai Y, Thomas GD, Yue Y, Yang HT, Li D, Long C (2009). Dystrophins carrying spectrin-like repeats 16 and 17 anchor nNOS to the sarcolemma and enhance exercise performance in a mouse model of muscular dystrophy. J Clin Invest.

[10] Kameya S, Miyagoe Y, Nonaka I, Ikemoto T, Endo M, Hanaoka K (1999). Alpha1-syntrophin gene disruption results in the absence of neuronal-type nitric-oxide synthase at the sarcolemma but does not induce muscle degeneration. J Biol Chem.

[11] Brenman JE, Chao DS, Xia H, Aldape K, Bredt DS (1995). Nitric oxide synthase complexed with dystrophin and absent from skeletal muscle sarcolemma in Duchenne muscular dystrophy. Cell..

[12] Chang WJ, Iannaccone ST, Lau KS, Masters BS, McCabe TJ, McMillan K (1996). Neuronal nitric oxide synthase and dystrophin-deficient muscular dystrophy. Proc Natl Acad Sci U. S A.

[13] Li D, Shin JH, Duan D (2011). iNOS ablation does not improve specific force of the extensor digitorum longus muscle in dystrophin-deficient *mdx*4cv mice. PLoS One.

[14] Tanihata J, Nagata T, Ito N, Saito T, Nakamura A, Minamisawa S (2018). Truncated dystrophin ameliorates the dystrophic phenotype of *mdx* mice by reducing sarcolipin-mediated SERCA inhibition. Biochem Biophys Res Commun.

[15] Shin J-H, Hakim C, Zhang K, Duan D (2011). Genotyping *mdx*, *mdx*3cv and *mdx*4cv mice by primer competition PCR. Muscle Nerve.

[16] Yoshida M, Hama H, Ishikawa-Sakurai M, Imamura M, Mizuno Y (2000). Biochemical evidence for association of dystrobrevin with the sarcoglycan-sarcospan complex as a basis for understanding sarcoglycanopathy. Hum Mol Genet.

[17] Imamura M, Nakamura A, Mannen H, Takeda S (2016). Characterization of WWP1 protein expression in skeletal muscle of muscular dystrophy chickens. J Biochem.

[18] Tanihata J, Suzuki N, Miyagoe-Suzuki Y, Imaizumi K, Takeda S (2008). Downstream utrophin enhancer is required for expression of utrophin in skeletal muscle. J Gene Med.

[19] Mikami Y, Kanemaru K, Okubo Y, Nakaune T, Suzuki J, Shibata K (2016). Nitric oxide-induced activation of the type 1 ryanodine receptor is critical for epileptic seizure-induced neuronal cell death. EBioMedicine..

[20] Ito N, Ruegg UT, Kudo A, Miyagoe-Suzuki Y, Takeda S (2013). Activation of calcium signaling through Trpv1 by nNOS and peroxynitrite as a key trigger of skeletal muscle hypertrophy. Nat Med.

[21] Louboutin JP, Rouger K, Tinsley JM, Halldorson J, Wilson JM (2001). iNOS expression in dystrophinopathies can be reduced by somatic gene transfer of dystrophin or utrophin. Mol Med.

[22] Gentil C, Le Guiner C, Falcone S, Hogrel JY, Peccate C, Lorain S (2016). Dystrophin threshold level necessary for normalization of neuronal nitric oxide synthase, inducible nitric oxide synthase, and ryanodine receptor-calcium release channel type 1 nitrosylation in Golden retriever muscular dystrophy dystrophinopathy. Hum Gene Ther.

[23] Xie QW, Cho HJ, Calaycay J, Mumford RA, Swiderek KM, Lee TD (1992). Cloning and characterization of inducible nitric oxide synthase from mouse macrophages. Science..

[24] Cho HJ, Xie QW, Calaycay J, Mumford RA, Swiderek KM, Lee TD (1992). Calmodulin is a subunit of nitric oxide synthase from macrophages. J Exp Med.

[25] Schmidt HH, Pollock JS, Nakane M, Forstermann U, Murad F (1992). Ca2+/calmodulin-regulated nitric oxide synthases. Cell Calcium.

[26] Béroud C, Tuffery-Giraud S, Matsuo M, Hamroun D, Humbertclaude V, Monnier N (2007). Multiexon skipping leading to an artificial DMD protein lacking amino acids from exons 45 through 55 could rescue up to 63% of patients with Duchenne muscular dystrophy. Hum Mutat.

[27] Aoki Y, Yokota T, Nagata T, Nakamura A, Tanihata J, Saito T (2012). Bodywide skipping of exons 45-55 in dystrophic *mdx*52 mice by systemic antisense delivery. Proc Natl Acad Sci U S A.

[28] Young CS, Hicks MR, Ermolova NV, Nakano H, Jan M, Younesi S (2016). A single CRISPR-cas9 deletion strategy that targets the majority of DMD patients restores dystrophin function in hiPSC-derived muscle cells. Cell Stem Cell.

[29] Nakamura A, Yoshida K, Fukushima K, Ueda H, Urasawa N, Koyama J (2008). Follow-up of three patients with a large in-frame deletion of exons 45-55 in the Duchenne muscular dystrophy (DMD) gene. J Clin Neurosci.

[30] Villalta SA, Nguyen HX, Deng B, Gotoh T, Tidball JG (2009). Shifts in macrophage phenotypes and macrophage competition for arginine metabolism affect the severity of muscle pathology in muscular dystrophy. Hum Mol Genet.

[31] Pastoret C, Sebille A (1995). *mdx* mice show progressive weakness and muscle deterioration with age. J Neurol Sci.

