# Supplementary figures and images for: iNOS is not responsible for RyR1 S-nitrosylation in mdx mice with truncated dystrophin
Source: BMC Musculoskelet Disord. 2020 Jul 21;21:479. doi: 10.1186/s12891-020-03501-0 (PMC7374827; doi:10.1186/s12891-020-03501-0)

Additional file 1

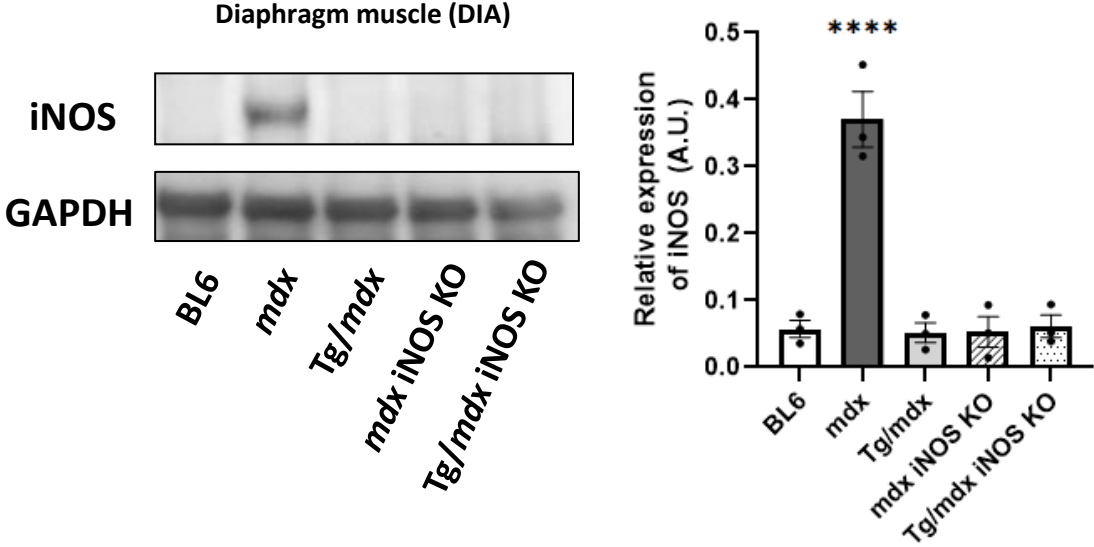

Supplement: Supplementary file 1 — Additional file 1 Protein expression of iNOS in DIA muscles. Western blots and quantification of iNOS in DIA muscles relative to the GAPDH. The original full blot with a loading control is shown in Additional file 2. Data are presented as means ± SEM. ****p < 0.0001 by ANOVA with Tukey-Kramer test (n = 3 mice per group). [file 12891_2020_3501_MOESM1_ESM.pdf]

Additional file 3

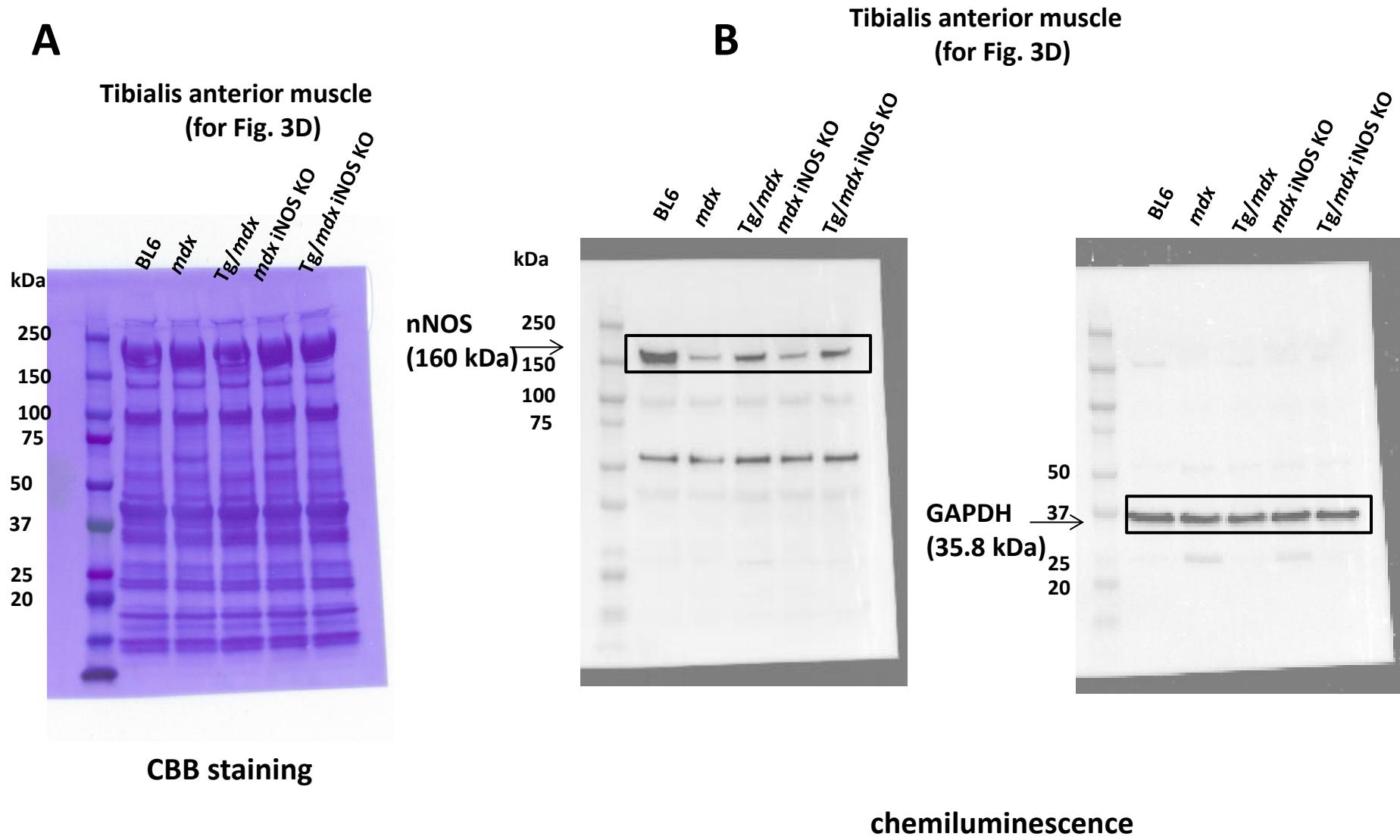

Supplement: Supplementary file 3 — Additional file 3. The original full blot of nNOS. (A) Whole image of PVDF membrane of Fig. 3d (nNOS expression in TA muscle) stained by Coomassie Brilliant Blue. The membrane was stained immediately after transferring. (B) Whole image of the immuno-Western blot of Fig. 3d. [file 12891_2020_3501_MOESM3_ESM.pdf]
