# Supplementary material for: iNOS is not responsible for RyR1 S-nitrosylation in mdx mice with truncated dystrophin
Source: BMC Musculoskelet Disord. 2020 Jul 21;21:479. doi: 10.1186/s12891-020-03501-0 (PMC7374827; doi:10.1186/s12891-020-03501-0)
Supplement: Supplementary file 2 — Additional file 2. The original full blot of iNOS for both tibialis anterior and diaphragm muscle. (A) Whole image of PVDF membrane of Fig. 3b (iNOS expression in TA muscle) and Additional file 1 (iNOS expression in DIA muscle) stained by Coomassie Brilliant Blue. The membrane was stained immediately after transferring. (B) Whole image of the immuno-Western blot of Fig. 3b and Additional file 1. [file 12891_2020_3501_MOESM2_ESM.pdf]

Additional file 2

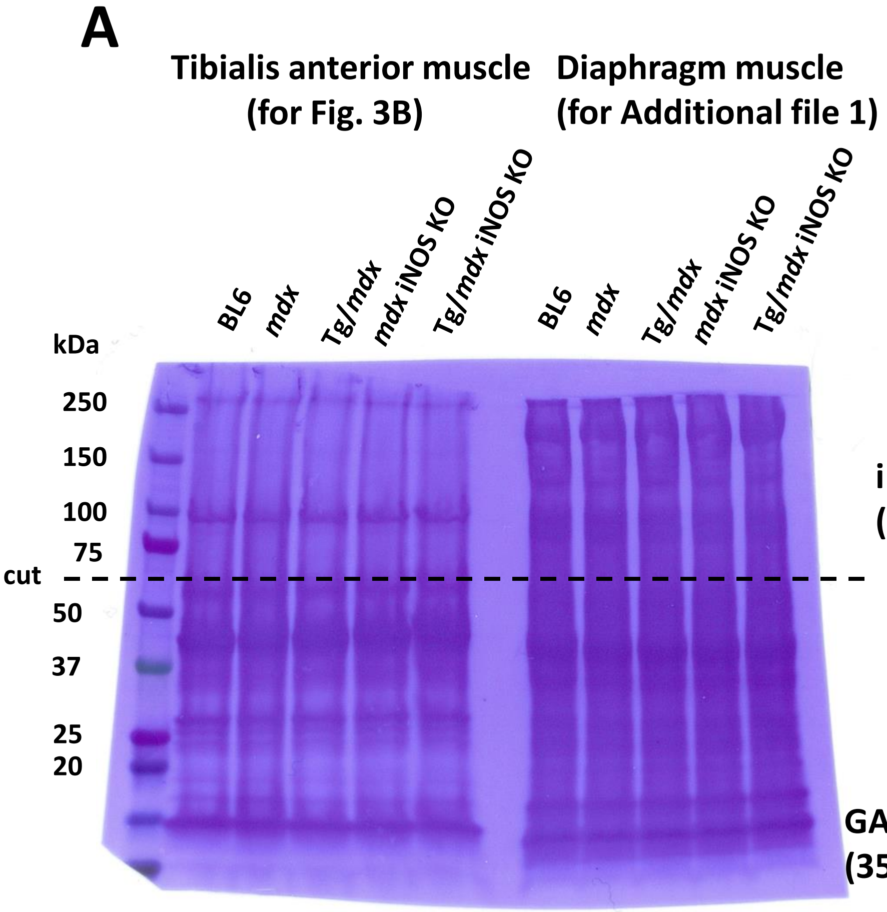

CBB staining

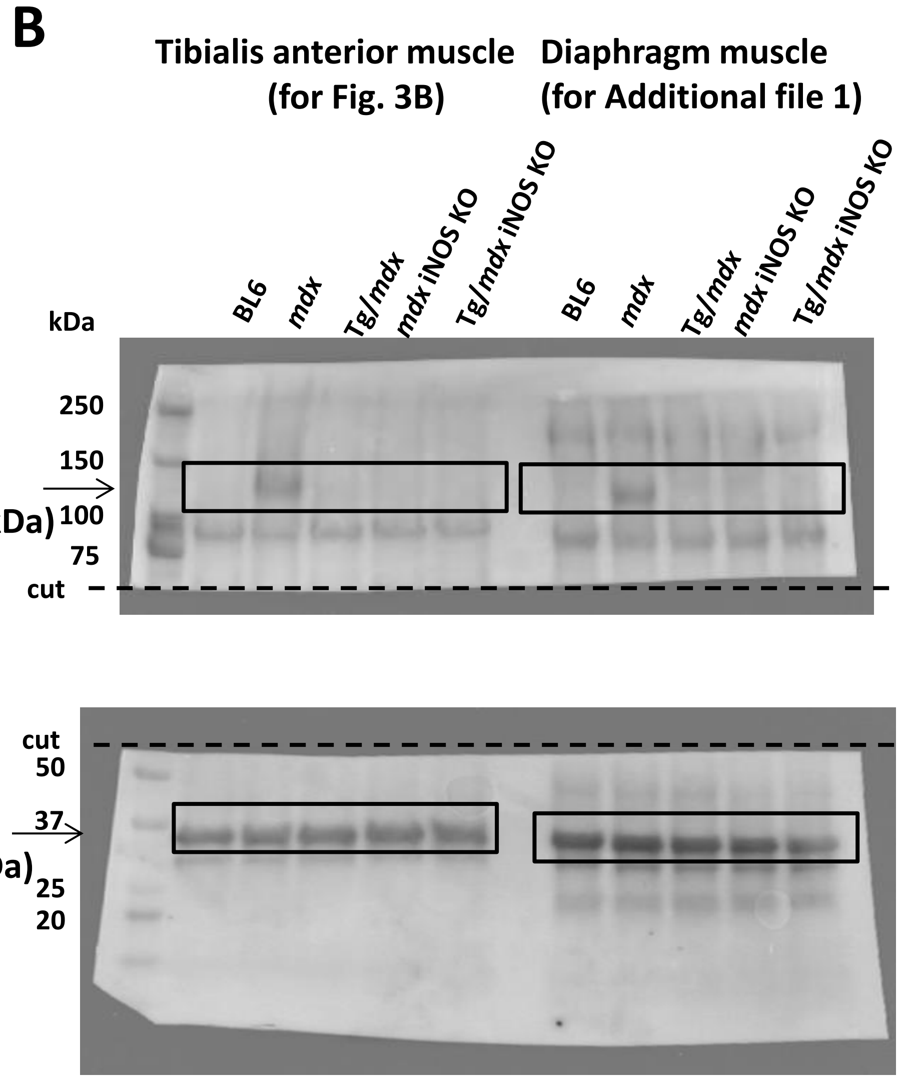

chemiluminescence
