# Supplementary material for: iNOS is not responsible for RyR1 S-nitrosylation in mdx mice with truncated dystrophin
Source: BMC Musculoskelet Disord. 2020 Jul 21;21:479. doi: 10.1186/s12891-020-03501-0 (PMC7374827; doi:10.1186/s12891-020-03501-0)

Additional file 4

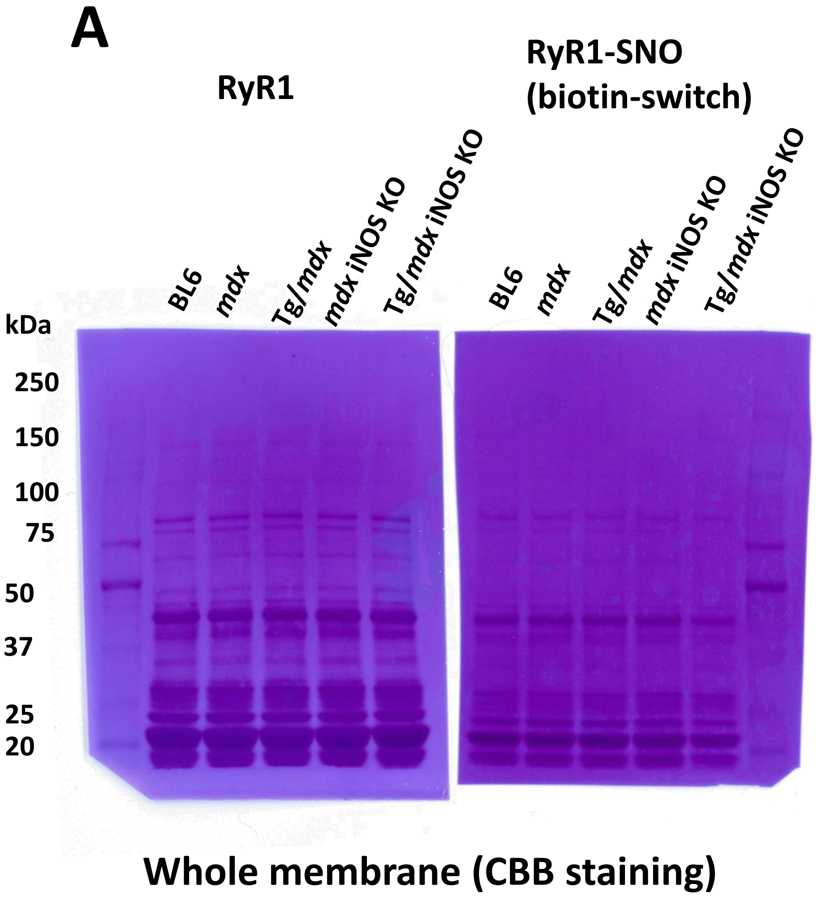

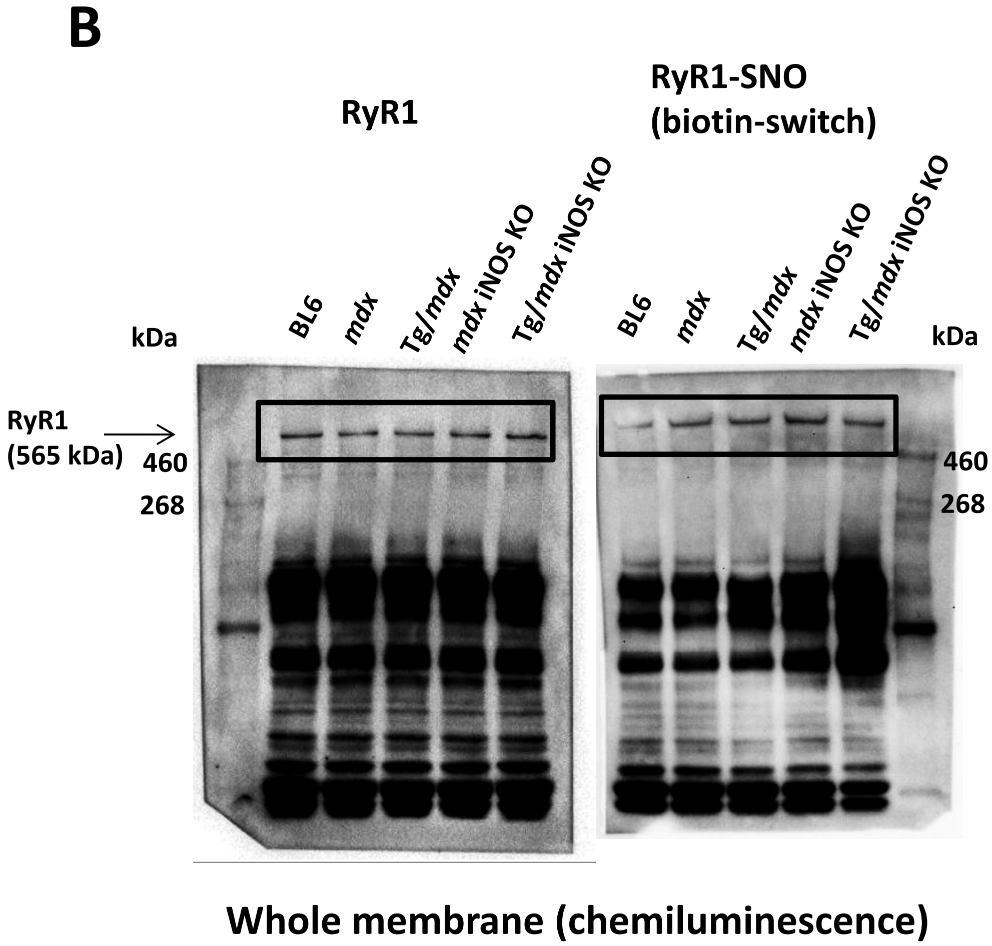

Supplement: Supplementary file 4 — Additional file 4. The original full blot of RyR1 and S-nitrosylated RyR1. (A) Whole image of PVDF membrane of Fig. 5a stained by Coomassie Brilliant Blue. The membrane was stained immediately after transferring. (B) Whole image of the immuno-Western blot of Fig. 5a. [file 12891_2020_3501_MOESM4_ESM.pdf]
